# The differential roles of the two NO-GC isoforms in adjusting airway reactivity

**DOI:** 10.1152/ajplung.00404.2021

**Published:** 2022-08-16

**Authors:** Malte Verheyen, Michelle Puschkarow, Stefanie Gnipp, Doris Koesling, Marcus Peters, Evanthia Mergia

**Affiliations:** ^1^Institute of Pharmacology and Toxicology, Ruhr-University Bochum, Bochum, Germany; ^2^Department of Experimental Pneumology, Ruhr-University Bochum, Bochum, Germany; ^3^Department of Molecular Immunology, Medical Faculty, Ruhr-University Bochum, Bochum, Germany

**Keywords:** airways, cGMP, guanylyl cyclase, nitric oxide, PDE5

## Abstract

The enzyme, nitric oxide-sensitive guanylyl cyclase (NO-GC), is activated by binding NO to its prosthetic heme group and catalyzes the formation of cGMP. The NO-GC is primarily known to mediate vascular smooth muscle relaxation in the lung, and inhaled NO has been successfully used as a selective pulmonary vasodilator. In comparison, NO-GC’s impact on the regulation of airway tone is less acknowledged and, most importantly, little is known about the issue that NO-GC signaling is accomplished by two isoforms: NO-GC1 and NO-GC2, implying the existence of distinct “cGMP pools.” Herein, we investigated the functional role of the NO-GC isoforms in respiration by measuring lung function parameters of isoform-specific knockout (KO) mice using noninvasive and invasive techniques. Our data revealed the participation and ongoing influence of NO-GC1-derived cGMP in the regulation of airway tone by showing that respiratory resistance was enhanced in NO-GC1-KOs and increased more pronouncedly after the challenge with the bronchoconstrictor methacholine. The tissue resistance and stiffness of NO-GC1-KOs were also higher because of narrowed airways that cause tissue distortion. Contrariwise, NO-GC2-KOs displayed reduced tissue elasticity, elastic recoil, and airway reactivity to methacholine, which did not even increase in an ovalbumin model of asthma that induced hyperresponsiveness in NO-GC1-KOs. In addition, conscious NO-GC2-KOs showed a higher breathing rate with a shorter duration of inspiration and expiration time, which remained faster even in the presence of bronchoconstrictors that slow down breathing. Thus, we provide evidence of two distinct NO/cGMP pathways in airways, accomplished by either NO-GC1 or NO-GC2, adjusting differentially the airway reactivity.

## Graphical abstract

**Figure:**
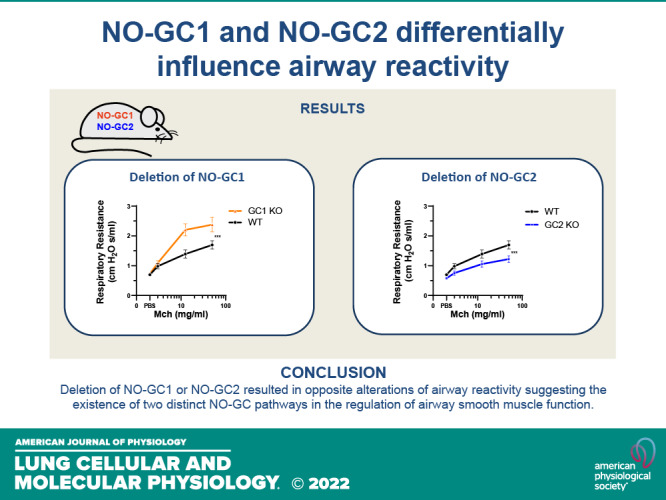

## INTRODUCTION

The heme-containing guanylyl cyclase (NO-GC; also referred to as sGC) catalyzes the formation of cGMP in response to NO binding, thereby mediating the transduction of the NO into the cGMP signal. A reduced heme iron (Fe^2+^) is important for NO binding to the enzyme’s prosthetic group, whereas an oxidized state (Fe^3+^) renders NO-GC insensitive to NO (1). However, the mechanisms regulating activation/deactivation of the NO-GC in vivo have not been fully elucidated (2).

The NO/cGMP signaling has been shown to participate in the regulation of many different functions, with the most well-known being the relaxation of vascular smooth muscle that adjusts the blood flow and tissue perfusion. In this regard, most of the NO-GC research in the lung, the organ with the highest NO-GC content, has been focused on pulmonary perfusion, and drugs that activate NO-GC, dependently or independently of NO, have been proven to treat pulmonary arterial hypertension (3). In comparison, participation of the NO/cGMP signaling in mediating airway relaxation is less unequivocal (4, 5). NO at normal airway concentrations has almost no effect in human airways in vitro and clinically is not a bronchodilator (6). Moreover, whereas inhaled NO applied in healthy subjects did not affect the airway tone, subsequent application of a β2-agonist caused bronchodilation (7). However, a recent study suggested that activation of NO-GC by drugs other than NO donors could be equi-effective in evoking bronchodilation in human small airways in situ, such as the activation of adenylyl cyclases by β2-adrenergic receptor agonists (8). It remains unclear, whether activation of NO-GC and cGMP generation could induce bronchodilation in healthy or diseased humans. Nevertheless, targeting NO-GC has been proposed as a potential bronchodilator strategy to treat obstructive lung diseases (9–11), and thus, further insights regarding NO-GC participation in respiratory lung function are of importance.

Under physiological conditions, the NO-GC in the lung is activated by NO that is mainly produced by the constitutively expressed NO synthases, eNOS, and nNOS. Expression of eNOS occurs predominantly in the endothelium and epithelium, and eNOS-derived NO has been suggested to regulate the adaptation of pulmonary perfusion to ventilation to assure the optimized ventilation/perfusion distribution (12, 13). Expression of nNOS is more restricted and mostly occurs in nonadrenergic, noncholinergic neurons (NANC) innervating airway smooth muscle cells (12). The NANC stimulation has been shown to induce airway relaxation of “in vitro” or “in situ” preparations and has been proposed to act as a defense against excessive airway smooth muscle contraction (14, 15). It should be mentioned here that the physiological influence of NO in the lung is not only exerted through the NO radical activating of the NO-GC but also through other oxidized forms of NO, such as *S*-nitrosothiols exerting the posttranslational modification and functional regulation of proteins (6).

The elevation in cellular cGMP is controlled in the following by one or more phosphodiesterase (PDE) enzymes that hydrolyze cGMP. In the lung, PDE5 is the PDE dominating cGMP-hydrolysis, and enhancing vascular cGMP by the pharmacological inhibition of PDE5 has been proved to be an effective treatment for pulmonary hypertension, similarly to the activation of NO-GC (16).

However, referring to NO-GC in the lung, one must consider that the common term NO-GC, in fact, comprises two distinct enzymes: NO-GC1 and NO-GC2, with comparable regulatory and enzymatic properties (17). Based on qPCR analyses among murine tissues, the lung has been identified as not only the organ with the highest amount of NO-GC1 (α_1_β_1_) but also the site where a high expression of NO-GC2 (α_2_β_1_) occurs (18). Data concerning the role of individual NO-GC isoforms are missing so far due to the lack of specific, biochemical tools (i.e., activators/inhibitors).

In the present study, we assessed the functional roles of the NO-GC1 and NO-GC2 isoforms in lung respiration by examining the lung function of knockout (KO) mice lacking either one of the enzymes. Compared with the deletion of both isoforms (19), isoform-specific KO mice do not exhibit an obvious phenotype and have a normal life expectancy (20). More importantly, genetic inactivation of one isoform does not lead to an altered expression of the second one (20, 21).

## MATERIALS AND METHODS

### Animals

Experiments were carried out with female NO-GC1 KO, NO-GC2 KO, and wild-type (WT) mice (10–25 wk old). The KO mice were generated and genotyped as described previously (20). Maintenance of the colony was achieved by backcrossing heterozygous (*NO-GC_a1_^+/−^* or *NO-GC_a2_^+/−^*) to WT mice (C57Bl/6Rj, Janvier). The resulting F1 compound heterozygotes were then intercrossed to generate F2 mice (KO and the respective WT strain). The KO and WT mice analyzed in the present study were either F1 littermates or F2 generation mice. The mice were held in a conventional mouse facility in a 12-h light-dark cycle at 22°C, 50%–60% humidity, and had access to food and water ad libitum. Mice were killed with cervical dislocation for the in vitro experiments. Experiments were approved by the animal ethics committee at the “Landesamt für Natur, Umwelt and Verbraucherschutz” of North Rhine-Westphalia, Germany (Approval Number: Az. 84.02.04.2013.A112), and performed according to the guidelines from Directive 2010/63/EU of the European Parliament on the protection of animals used for scientific purposes.

### Preparation of Lung Homogenates for Enzymatic Activity Assays

The middle lobe of the lungs was homogenized on ice in 10 volumes of homogenization buffer [triethanolamine (TEA)/HCl 50 mM, NaCl 50 mM, EDTA 1 mM, DTT 2 mM, benzamidine 0.2, phenylmethylsulfonyl fluoride 0.5 and pepstatin A 1 µM, pH 7.4, 4°C] using a glass/glass Potter‐Elvehjem homogenizer (1,000 rpm, 2 min). The protein content was determined in triplicate by using the Bradford method (Bio‐Rad). Thereafter, aliquots of the homogenates were used to measure NO-GC and PDE activity.

### Measurement of NO-GC Activity

The NO-stimulated GC activity was determined in aliquots of the homogenates (5–10 µg) by the addition of 100 µM 2-(*N*,*N*-diethylamino)-diazenolate-2-oxide (DEA-NO; Enzo) in the presence of GTP (0.25 mmol/L) for 10 min at 37°C. Reactions were performed in triplicate and terminated by removing an aliquot (10 µL) into ice-cold radioimmunoassay (RIA) buffer (90 µL: 100 mM CH_3_COONa; pH 6.0) and freezing immediately at −20°C. The cGMP formed was detected by RIA in duplicate, as described previously (20, 22).

### PDE Assay

The PDE activity was measured in aliquots of the homogenates (5–10 µg) by conversion of [^32^P]cGMP to guanosine and [^32^P]phosphate in the presence of alkaline phosphatase (Sigma) at 37°C for 5 min, as detailed previously (20). Homogenates were assayed in triplicate for total PDE and tadalafil-inhibited PDE activity in the absence or presence of 10 µM tadalafil, respectively. The PDE5 activity was obtained by subtracting the tadalafil-inhibited activity from the total activity, and was found not to differ between the genotypes.

### Determination of cGMP Content in Lung Slices

Lung slices of 250 µm were cut from the left lobe in the dorsal-ventral plane with a tissue chopper (*McIlwain*) and then selected to be completely occupied by lung tissue and not contain large bronchi, vessels or other miscellaneous tissue. Slices were equilibrated for 40 min in temperate (37°C), oxygenated (in 95% O_2_, 5% CO_2_) Krebs–Henseleit buffer in the presence of l-NAME (200 µM) to exclude any effects of endogenous NO formation. The PDE inhibitors (PDE5 inhibitors: sildenafil 100 µM, or tadalafil 100 µM; PDE1 inhibitor: 8-MMX, 200 µM), when used, were added for the last 20 min. The increase of cGMP was induced by adding *S*-nitroso-glutathione (GSNO 100 µM) for an additional 5 min. Reactions were terminated by snap freezing the slices in liquid nitrogen. Thereafter, slices were homogenized in 70% (vol/vol) ice-cold ethanol using a glass/glass homogenizer, and then centrifuged (14,000 *g*, 15 min, 4°C). Supernatants were dried at 95°C and their cGMP content was measured by RIA in duplicate. Protein pellets were dissolved in 0.1 M NaOH-0.1% SDS, and the protein content was determined using the bicinchoninic acid method (Thermo Scientific). The IC_50_ value of sildenafil for PDE5 is 8.5 nM and for PDE1 350 nM. The IC_50_ value of tadalafil for PDE5 is 9.4 nM and for PDE1 > 10,000 nM. The IC_50_ value of 8-MMX (8-methoxymethyl 3-isobutyl-1-methylxanthine) for PDE1 is 5.2 µM.

### Invasive Lung Function Measurements

Mechanical properties of the lungs were assessed using the forced oscillation technique applied by the Flexivent FX1 apparatus (SCIREQ, Montreal, QC, Canada), which was operated by flexiWare 7.6 software. Mice were anesthetized (ketamine 125 mg/kg and xylazine 16 mg/kg), spontaneous breathing was suppressed with 0.8 mg/kg pancuronium, and then they were cannulated (tracheostomy with ligation) and ventilated with a tidal volume of 10 mL/kg at a breathing frequency of 150 breaths/min and a positive end-expiratory pressure of 3 cmH_2_O. After connection to the ventilator, the lung was inflated twice to 27 cmH_2_O over 3 s and held at that pressure for an additional 3 s (deep inflation) to recruit the lung beyond any closed airway and to standardize the lung volume history. Following deep inflation, three different perturbations were applied: *1*) pressure-volume loops with stepwise increasing pressure (PVs-P) from 3–30 cmH_2_O and the volume-driven maneuvers; *2*) SnapShot (1.2 s, 2.5 Hz); and *3*) Quick Prime-3 (3 s, 1–20.5 Hz). The running script included a sequence of SnapShot/QuickPrime-3/PVs-P/Deep inflation that was repeated three times to determine the parameters at baseline. An additional sequence was included consisting of alternating SnapShot and QuickPrime-3 perturbations that were applied and repeated 12 times for a period of ∼4 min following the activation of the nebulizer to assess the airway responsiveness to inhaled methacholine (0, 3, 12.5, and 50 mg/mL). Only measurements with a coefficient of determination > 0.90 were accepted and used in the data analysis. Data obtained with the SnapShot perturbation were analyzed by the software using the single-compartment model to determine the total respiratory resistance (*R*_rs_) and elastance (*E*_rs_). Data from Quick Prime-3 perturbation were analyzed using the constant-phase model, which uses an equation with four parameters and allows one to distinguish the airway (*R*_n_, Newtonian resistance) and the lung tissue (*G*, tissue damping; *H*, tissue elasticity) contributions. Static compliance (*C*_st_) was calculated directly from the deflating arm of the PV loop between pressures of 3–7 cmH_2_O. An estimate of inspiratory capacity (parameter A) and the shape parameter *K* was calculated by the software by fitting the PV curves into the Salazar–Knowles equation. Baseline values produced by our protocol and the operator were comparable to previously published values for C57BL/6 mice (23, 24).

### Lung Morphometry

Lungs were fixed in situ via intratracheal instillation of 4% paraformaldehyde at a pressure of 25 cmH_2_O. After the trachea was tied off with a ligature, the lungs were placed in a falcon tube containing the fixative for 4 d (4°C) before processing. After dehydration in a graded ethanol series followed by xylene, the right lung was embedded in paraffin and sectioned in the dorsal-ventral plane. Sections (3 µm) were obtained at 0, 200, and 400 µm; 0 was defined as the level where the mainstem bronchus was observed. Two consecutive slices of each level were placed onto charged slides. Slices were then stained with Sirius red and imaged at ×2, ×20, and ×40 objective using an Olympus BX61 (Hamburg, Germany). Quantification of the mean linear intercept was performed using ImageJ (25) and the protocol developed by Crowley et al. (26). Six image fields per section were selected and quantified using horizontal and vertical test lines by a blinded investigator. In summary, six sections (i.e., 36 image fields) per animal were examined.

### Noninvasive Whole Body Plethysmography

Measurements of respiratory parameters in conscious, unrestrained mice were performed using four-chamber whole body plethysmography (Buxco electronics, now DSI). Chambers were calibrated with 1.0 mL air injection, according to the manufacturer’s instructions, and then mice were placed into the chamber plethysmograph (400 mL) and allowed 5 min to acclimate before recordings started. Mice were monitored over a period of 5 min for basal measurements and then exposed to a 2-min aerosolization of increasing doses of methacholine (0, 6.25, 12.5, 25, and 50 mg/mL) or serotonin (0, 2.5, 5, 10, and 15 mg/mL), followed by 3 min additional recording. The following ventilatory parameters were determined by the software (Biosystem XA) and used for analysis: respiratory rate (*f*), time of inspiration (*T*_i_), time of expiration (*T*_e_), peak inspiratory flow (PIF), peak expiratory flow (PEF), tidal volume (*T*_v_), minute ventilation (*M*_v_) and Penh (enhanced pause). KO and strain-matched WT mice were measured in parallel. In addition, we ensured that the Penh parameter in our mice strains correlates empirically with the results of the invasive measurements by measuring subsets of the mice with the Buxco apparatus and with the Flexivent system in the following.

### Immunization Protocol

Ovalbumin (OVA) emulsified with Imject Alum [(Al (OH)_3_:Mg(OH)_2_] was administered intraperitoneally (200 µL of 100 µg OVA/mL ip) on *days 0*, *14*, and *21*, and subsequently via aerosol inhalation (1% OVA in PBS for 30 min) on two consecutive days of every week for 12 wk. The OVA aerosol was generated using a PARI-Boy aerosol generator (PARI, Starnberg, Germany). Control mice were sensitized similarly with intraperitoneal injections of OVA, but were challenged with equivalent volumes of PBS. Airway responsiveness to inhaled Mch was measured 24 h after the last challenging dose of OVA or PBS.

### Bronchoalveolar Lavage

Three days after the last OVA challenge, lungs were lavaged via tracheal cannula with 2× 1 mL PBS, and the leukocytes in the bronchoalveolar lavage (BAL) fluid (BALF) were counted. Cytospin slides were prepared from the remaining BALF and stained with hematoxylin and eosin (HAEMA – Quick Stain LT Sys, Labor+Technik), according to the manufacturer’s instructions. Specific immune cells (macrophages, lymphocytes, eosinophils, and neutrophils) were identified as previously (27) and counted by a blinded investigator. The cell numbers were normalized to the total cell count. A number of at least 300 cells per sample was differentially determined.

### Statistical Analysis

**Figure 1.**
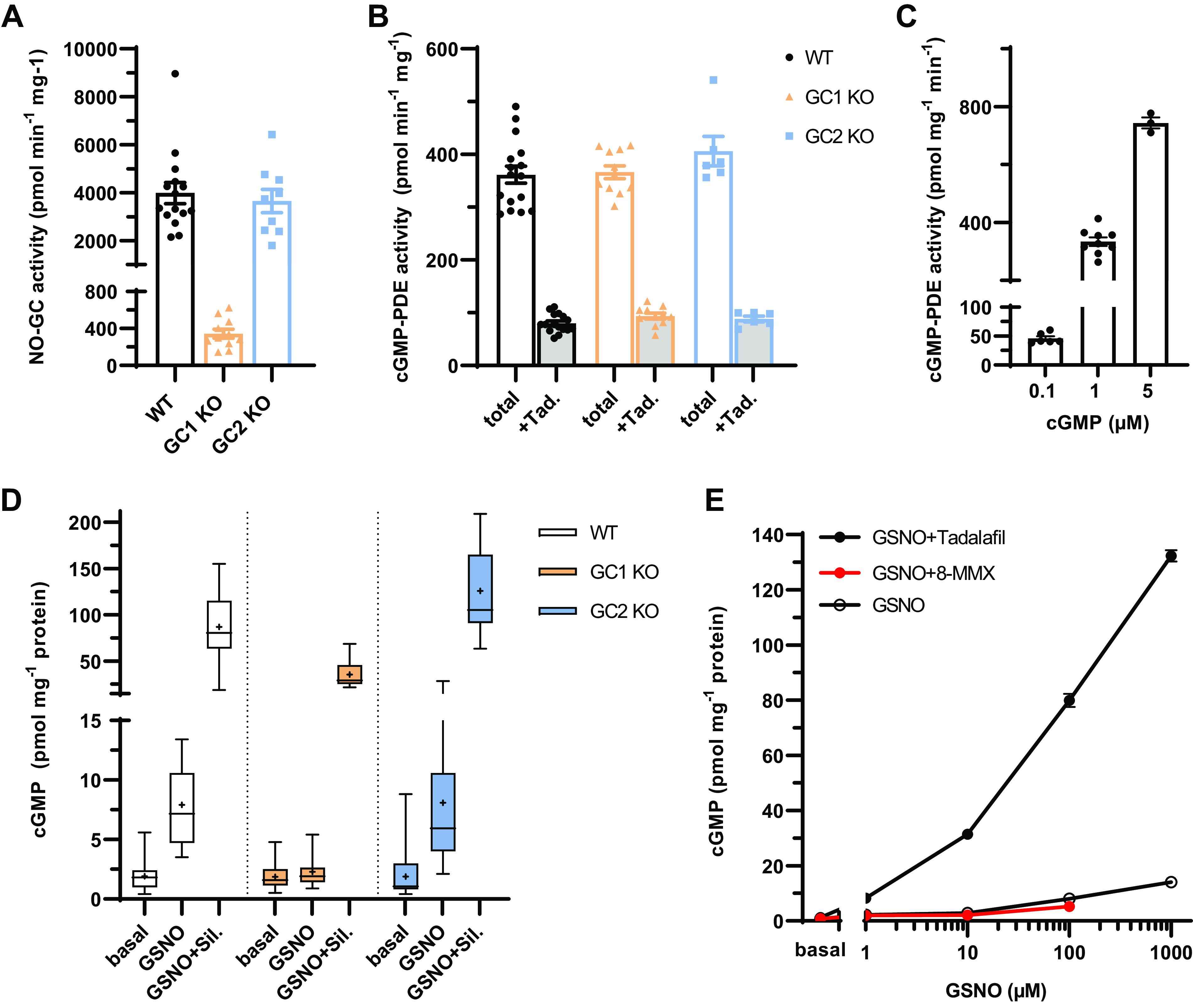
Contribution of NO-GC1 and NO-GC2 to cGMP formation in lung parenchyma; impact of PDE5. *A* and *B*: NO-induced cGMP formation (100 µM DEA-NO, 10 min) (*A*) and cGMP degradation at 1 µM cGMP (*B*) as the substrate in the absence or presence of a PDE5 inhibitor (tadalafil 10 µM) were measured in lung homogenates of WT (*n* = 16–15), NO-GC1 KO (*n* = 11), and NO-GC2 KO (*n* = 9–6) mice. *C*: cGMP degradation in lung homogenates of WT mice by different substrate concentrations [0.1 µM (*n* = 6), 1 µM (*n* = 9), and 5 µM (*n* = 3)]. *D*: increases of cGMP in lung slices of WT, NO-GC1 KO, and NO-GC2 KO mice measured under nonstimulated or stimulated conditions (GSNO 100 µM, 5 min ± sildenafil 100 µM, 20-min preincubation). Box and whiskers from minimum to maximum with mean annotated as “+” from six WT, five NO-GC1 KO, and six NO-GC2 KO mice with *n* = number of slices (nonstimulated: 40, 19, 24; GSNO-stimulated: 19, 20, 20, and GSNO+sildenafil-stimulated: 20, 20, 18 slices) are shown. *E*: increase of cGMP in WT lung slices in response to stimulation (5 min) with different GSNO concentrations (1, 10, 100, and 1,000 µM) under PDE5- (100 µM tadalafil, 20 min preincubation) or PDE1-inhibited conditions (200 µM 8-MMX, 20-min preincubation). Averaged value means ± SE of 7–13 slices from six WT mice are shown, with the exception of the condition at 1 mM GSNO and GSNO + 8-MMX, where only four of the mice are represented. Statistical comparisons in *A*, *B*, and *D* were performed using the Kruskal–Wallis test with Dunn’s correction. Comparison revealed no significance between PDE activities among the genotypes (*B*) or cGMP values induced by GSNO+Sil (*D*). GSNO, *S*-nitroso-glutathione; KO, knockout; NO-GC, nitric oxide-sensitive guanylyl cyclase; PDE, phosphodiesterase; Sil, sildenafil; Tad, tadalafil; WT, wild type; 8-MMX, 8-methoxymethyl 3-isobutyl-1-methylxanthine.

All experimental procedures were performed by analyzing KO and WT mice in parallel. Values reported represent means ± SE, except in Fig. 1*D*, where box and whiskers plots are shown. Statistical analyses were performed using GraphPad Prism 9.2.0 (GraphPad Software, San Diego, CA). The experimental results were evaluated a priori using the D’Agostino-Pearson normality test for Gaussian distribution. Differences between groups with normal distribution were analyzed using one-way analysis of variance (ANOVA) with Fisher’s least significant difference (LSD) test, two-way ANOVA, or Student’s *t* test. The Kruskal–Wallis test with Dunn’s correction was used for nonparametric analyses. The exact test that was used and the *n* numbers are indicated in the legend of each graph. Significance levels are indicated as follows: *P < 0.05, **P < 0.01, and ***P < 0.001.

## RESULTS

### Contribution of NO-GC1 and NO-GC2 Isoforms to the cGMP Signals in Lung Parenchyma

Both NO-GC isoforms contribute to NO-induced cGMP formation in the lung, as shown by the NO-GC activity measured in homogenates deficient in one isoform (NO-GC1 KO, NO-GC2 KO, and WT; Fig. 1*A*). The cGMP signal is tightly controlled by cGMP degradation; therefore, we also measured the cGMP-hydrolyzing activity using 1 µM cGMP as the substrate and in the absence or presence of tadalafil (10 µM) to measure PDE5. The results confirmed the expectation that PDE5 constitutes the major part of the cGMP degradation in the lung (77%). However, despite the absence of one NO-GC isoform, the cGMP-degrading activity did not differ between the genotypes (Fig. 1*B*). However, the cGMP-degrading activity is less in the presence of a lower cGMP concentration, as degradation depends on the *K*_m_ value of a particular PDE and, thus, an adjustment of the expression level of this particular PDE is not required (Fig. 1*C*).

Lung slices containing mainly parenchyma were incubated with GSNO (100 µM, 5 min) in the presence or absence of the PDE5 inhibitor sildenafil (100 µM) to judge the contribution of the isoforms to cellular cGMP signals. Similar NO-induced cGMP increases were detected in WT and NO-GC2 KO slices, whereas NO did not increase cGMP in NO-GC1 KO (Fig. 1*D*). The inhibition of PDE5 resulted in an additional 10-fold increase of cGMP comparable between the genotypes. These results clearly reveal a contribution of NO-GC2 to NO-induced cGMP formation in the lung parenchyma.

The GSNO (100 µM) induced a similar cGMP increase in WT slices in the presence of tadalafil (100 µM), the same as with sildenafil (100 µM), providing independent evidence that cGMP signals in lung parenchyma are controlled by PDE5 (Fig. 1*E*). In addition, the PDE1 inhibitor (8-MMX, 200 µM) did not enhance the cGMP increase, excluding a participation of PDE1 (similar *K*_m_ to PDE5) under physiological conditions.

### NO-GC1 and NO-GC2 KO Mice Exhibit Altered Functional Properties of Lungs

**Figure 2.**
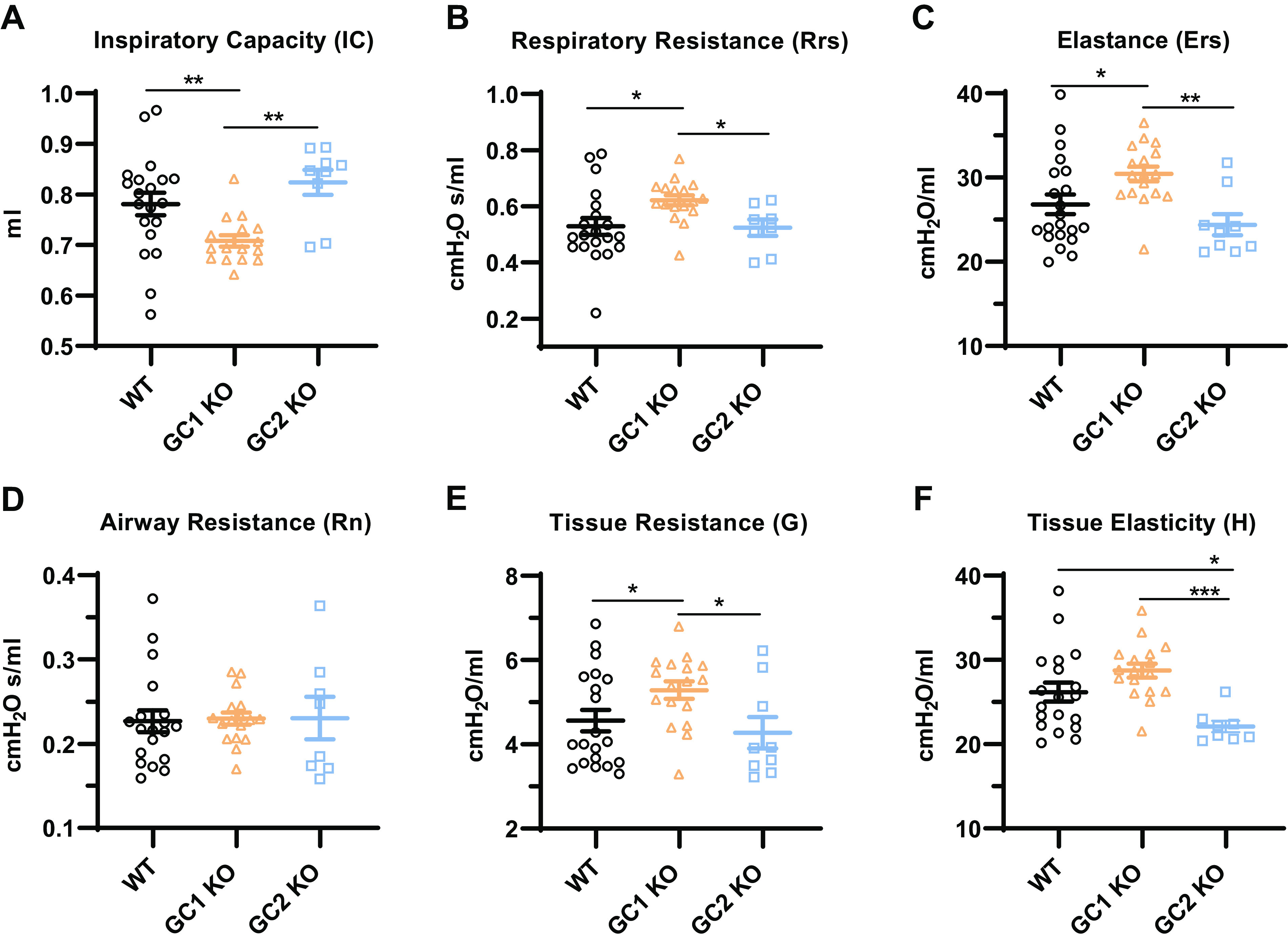
Airway function of NO-GC1 and NO-GC2 KO mice at baseline. Respiratory parameters in tracheotomized WT and KO mice were assessed by applying the following perturbations using the FlexiVent system (SCIREQ). Deep inflation was performed to measure inspiratory capacity (*A*), snapshot perturbation to calculate parameters, such as respiratory resistance (*B*) and elastance (*C*), and prime-3 perturbation to differentiate between airway resistance (*D*) and tissue parameters, such as tissue resistance (*E*) and elasticity (*F*). The average of three correct maneuvers per mouse are shown, along with the group means ± SE. The *y*-axis is truncated. Measurements were performed with *n* = 21 WT, 17 GC1KO and 9 GC2KO mice; missing values are because of low coefficients of determination in the data analysis. Statistical significance was determined by one-way ANOVA with Fisher’s LSD test. ***P < 0.001, **P < 0.01, and *P < 0.05. KO, knockout; LSD, least significant difference; NO-GC, nitric oxide-sensitive guanylyl cyclase; WT, wild type.

We measured the respiratory mechanics in tracheotomized WT and KO mice using the FlexiVent system to study the participation of the NO-GCs in the functional properties of the lung. Inspiratory capacity (Fig. 2*A*) in response to the deep inflation maneuver was reduced in NO-GC1 KOs, suggesting restricted or less lung volumes in these mice. A snapshot perturbation analysis revealed a higher respiratory resistance of NO-GC1 KOs, in agreement with the role of NO-GC1 in bronchial smooth muscle relaxation (Fig. 2*B*). In addition, NO-GC1 KOs showed an increase of elastance (and a corresponding decrease of compliance), which points to an altered elasticity of the lung (Fig. 2*C*). Compared with NO-GC1 KOs, lungs of NO-GC2 KO mice showed WT-like properties (Fig. 2, *A–C*).

Unlike the snapshot perturbation in which the lung is seen as a single compartment (lung and chest wall), the prime-3 perturbation considers the lung as a multiple compartment and allows one to differentiate between the airways and lung tissue (Fig. 2, *D–F*). Interestingly, airway resistance at the baseline did not differ between the genotypes, while tissue parameters did. Consistent with an increase in tissue stiffness, NO-GC1 KO mice displayed elevated *G* values compared with WT and NO-GC2 KOs (tissue resistance; Fig. 2*E*). By contrast, *H* values of NO-GC2 KOs were decreased compared with WT and NO-GC1 KOs (tissue elasticity; Fig. 2*F*), pointing to a decrease in the elastic recoil.

**Figure 3.**
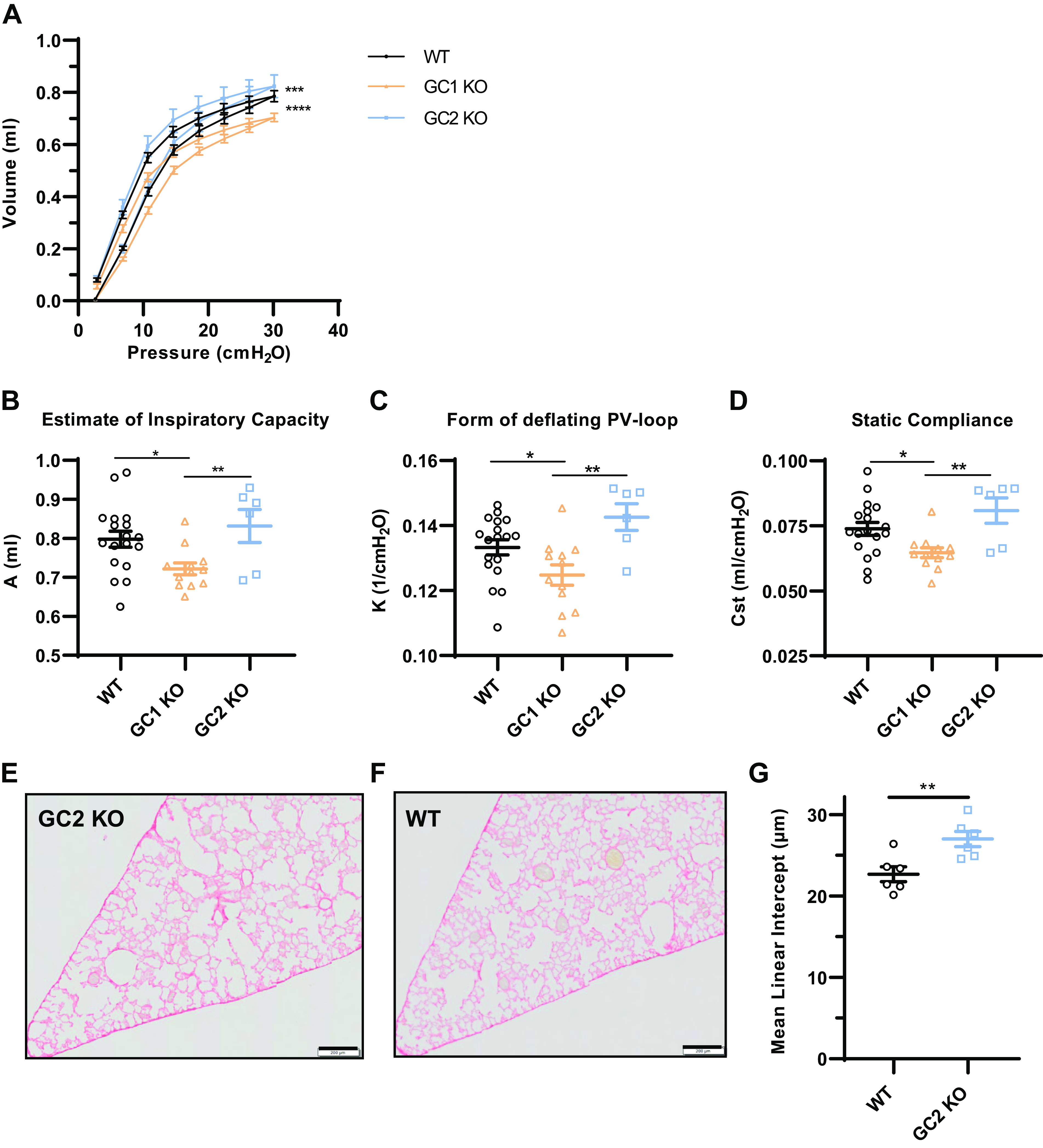
Effect of NO-GC1 or NO-GC2 deletion on baseline elastic properties of the respiratory system. Lung function measurements of WT (*n* = 18), GC1KO (*n* = 12), and GC2KO mice (*n* = 6). *A*–*D*: pressure-volume curves were performed in WT, NO-GC1, and NO-GC2 KO mice using the FlexiVent system (*A*) and used to obtain an estimate of inspiratory capacity (*B*), the shape constant *K* (*C*), and static compliance (*D*). The averages of three correct maneuvers per mouse are shown along with the group means ± SE. The *y*-axis is truncated in *B*, *C*, and *D*. Lung tissue sections from GC2 KO (*E*) and strain-matched WT (*F*) lungs fixed at a pressure of 25 cmH_2_O. Sections were stained with Sirius red (scale bar: 200 µm). *G*: mean linear intercept quantification of six mice per genotype. Statistical significance was determined by two-way ANOVA (*A*), one-way ANOVA with Fisher’s LSD test (*B*–*D*), and two-sided Student’s *t* test (*G*). ****P < 0.0001, ***P < 0.001, **P < 0.01, and *P < 0.05. KO, knockout; LSD, least significant difference; NO-GC, nitric oxide-sensitive guanylyl cyclase; PV, pressure-volume; WT, wild type.

In accordance with these findings, by applying the PV-loop perturbation, pressure-volume curves of NO-GC1 KOs showed a downward shift, whereas those of the NO-GC2 KOs displayed an upward shift (Fig. 3*A*). Similar to inspiratory capacity (Fig. 2*A*), the estimated inspiratory capacity derived from the PV curves of NO-GC1 KOs was also reduced (*A*; Fig. 3*B*). In addition, the shape constant *K* (Fig. 3*C*) and static compliance (*C*_st_; Fig. 3*D*) of NO-GC1 KOs decreased compared with WT and NO-GC2 KOs, which again is compatible with an increased elastic recoil. It is notable that the corresponding values in NO-GC2 KOs tended to be higher, even though they did not reach significance.

We next quantified the mean linear intercept (MLI), i.e., the mean free distance in the acinar airspace complex, in lung sections of NO-GC2 KO and WT mice (Fig. 3, *E–G*). Consistent with the pulmonary mechanics, the MLI was increased from 22.7 µm for WT to 27.02 µm for the NO-GC2 KO mice (19 ± 4%), indicating an alveolar enlargement in the KO lungs.

### Lungs of NO-GC1 and NO-GC2 KO Mice Respond Differently to Provocation with Methacholine

**Figure 4.**
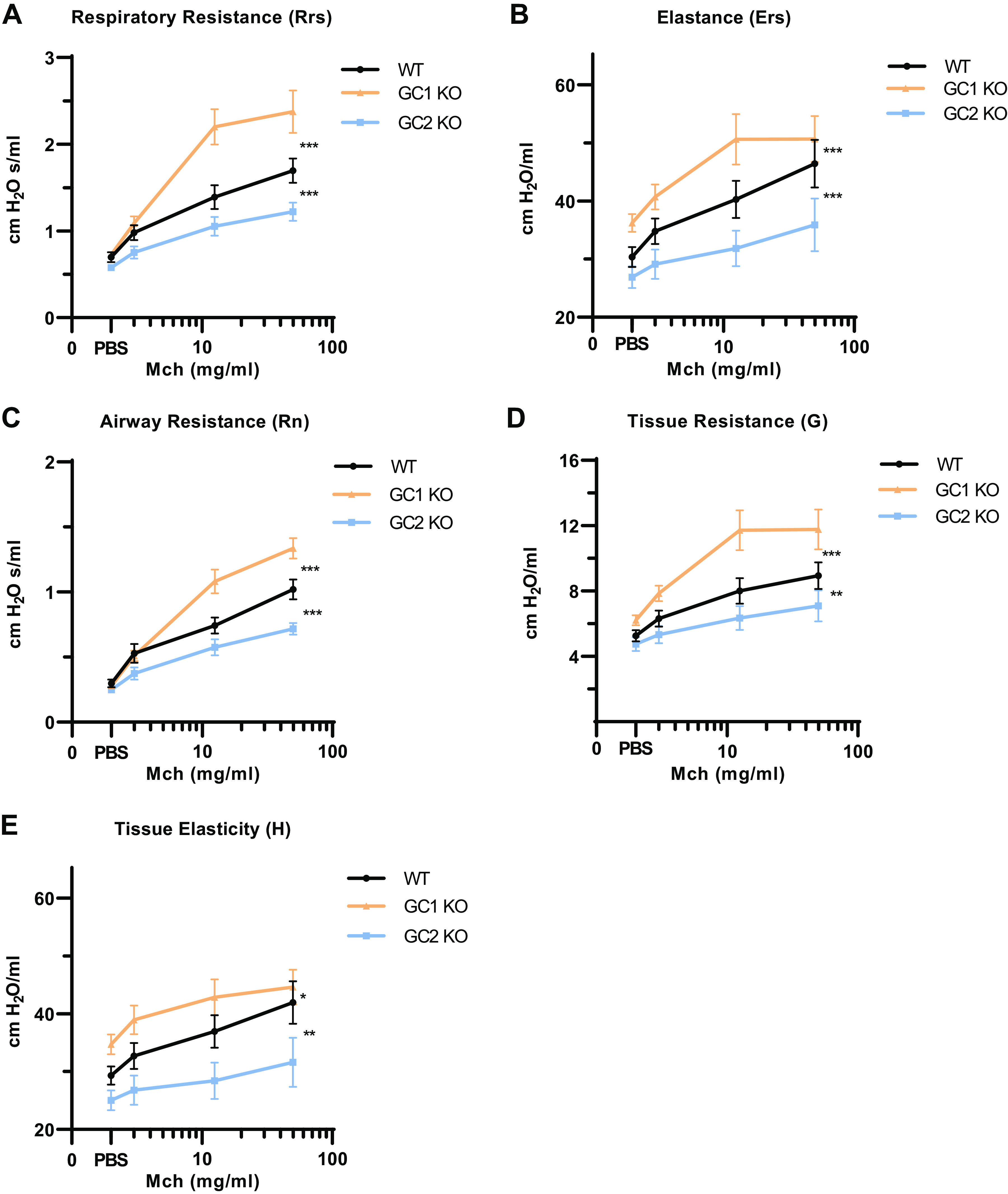
Airway function of NO-GC1 and NO-GC2 KO mice in response to methacholine. The airway reactivity of WT, NO-GC1, and NO-GC2 KO mice to increasing concentrations of Mch (0, 3, 12.5, and 50 mg/mL) was measured by the FlexiVent perturbation snapshot to obtain respiratory resistance (*A*) and elastance (*B*), and prime-3 to calculate airway resistance (*C*), tissue resistance (*D*), and tissue elasticity (*E*). The means ± SE of *n* = 16 WT, 11 GC1KO, and 9 GC2KO mice are shown. The average of each point was calculated from the peak value for each parameter of 12 maneuvers applied per mouse and the drug concentration. The *y*-axis is truncated in *B* and *E*. Statistical significance was determined by two-way ANOVA. ***P < 0.001, **P < 0.01, and *P < 0.05. KO, knockout; Mch, methacholine; NO-GC, nitric oxide-sensitive guanylyl cyclase; PBS, phosphate-buffered saline; WT, wild type.

After recording the baseline lung mechanics, methacholine (Mch), a muscarinic agonist, was administered by nebulization to induce airway smooth muscle contraction. The concentration-dependent increase of respiratory (*R*_rs_) and airway resistance (*R*_n_) induced by Mch was clearly higher in NO-GC1 KOs than in WT mice but lower in NO-GC2 KOs (Fig. 4, *A* and *C*). Comparable patterns were found for the tissue parameters (i.e., *E*_rs_, *G*, and *H*), which increased in the course of airway constriction (Fig. 4, *B, D*, and *E*). Overall, whereas the NO-GC1 KOs revealed hyperresponsiveness to Mch, the response of the NO-GC2 KOs was reduced.

Administration of a bronchodilator, salbutamol (2 mg/mL), concomitant with Mch (50 mg/mL) was able to reduce the higher increase of the airway resistance in the NO-GC1 KOs to WT level (Supplemental Fig. S1; all Supplemental Material is available at https://doi.org/10.6084/m9.figshare.19771723).

### Conscious KO Mice of NO-GC1 or NO-GC2 Display Different Alterations in Respiratory Parameters

**Figure 5.**
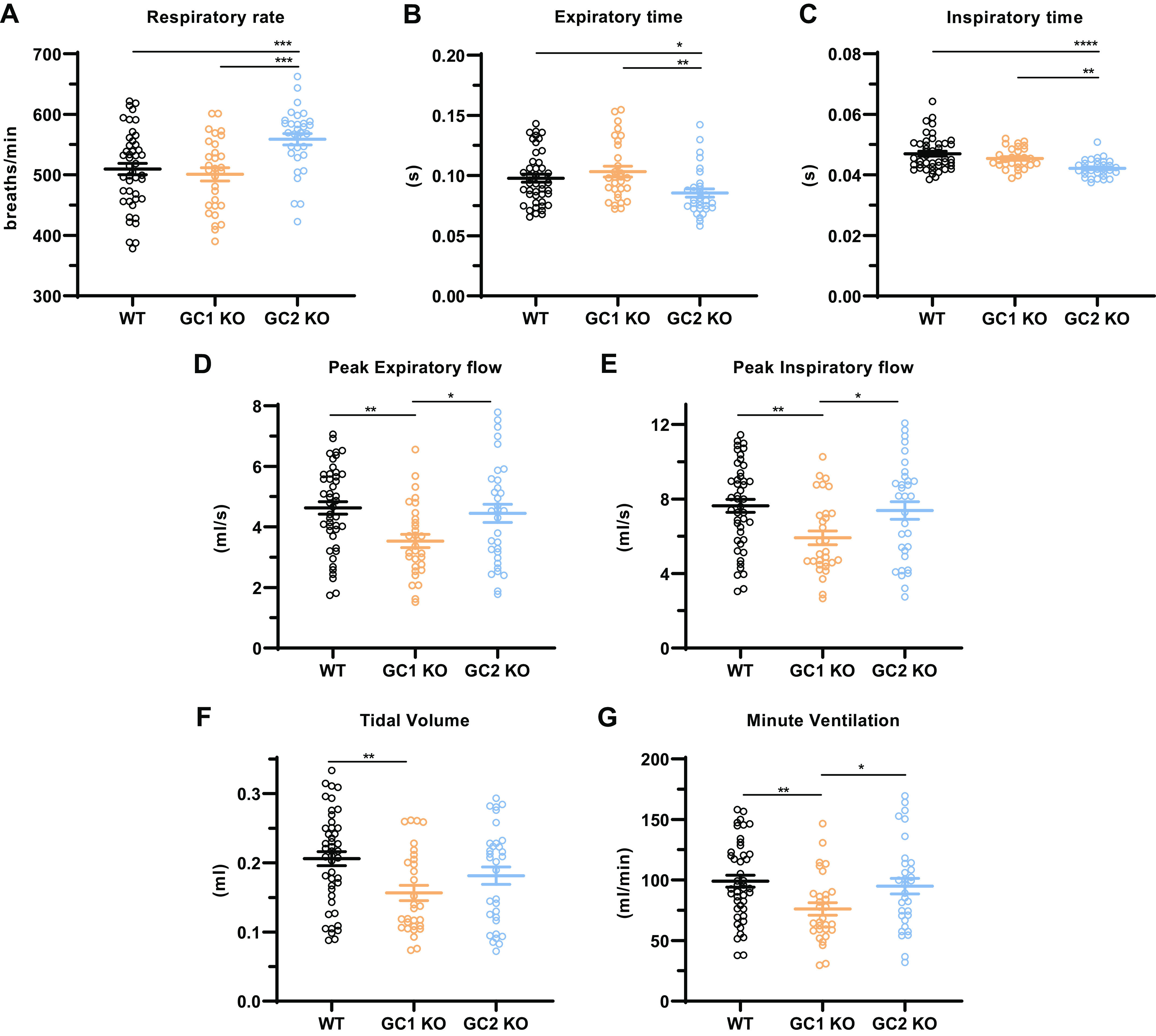
Baseline respiratory parameters of conscious NO-GC1 and NO-GC2 KO mice. Respiratory parameters of conscious WT, NO-GC1, and NO-GC2 KO mice were assessed by unrestrained whole body plethysmography. The respiratory rate (*A*), time of expiration (*B*) and inspiration (*C*), peak expiratory (*D*) and inspiratory flow (*E*), tidal volume (*F*), and minute ventilation (*G*) are represented. Individual values of 45 WT, 33 NO-GC1 KO, and 32 NO-GC2 KO mice along with the group means ± SE are shown. The individual values represent the average of values collected over 5 min. The *y*-axis is truncated in *A*. Statistical significance was determined by one-way ANOVA with Fisher’s LSD test. ****P < 0.0001, ***P < 0.001, **P < 0.01, and *P < 0.05. KO, knockout; LSD, least significant difference; Mch, methacholine; NO-GC, nitric oxide-sensitive guanylyl cyclase; PBS, phosphate-buffered saline; WT, wild type.

We also examined conscious mice using unrestrained single-chambered whole body plethysmography to gain further insights into NO-GCs’ function in the lung. Analysis of the plethysmographic recordings in NO-GC2 KOs revealed an enhanced respiratory rate at rest (Fig. 5*A*), paralleled by a shortened time of expiration (Fig. 5*B*) and inspiration (Fig. 5*C*), whereas the phenotype of NO-GC1 KO mice was WT-like. Conversely, lung flows, tidal volume, and minute ventilation in NO-GC1 KOs were found to be reduced at rest, whereas the respective parameters in NO-GC2 KOs were WT-like (Fig. 5, *D–G*).

### Changes of Respiratory Parameters in Conscious NO-GC1 and NO-GC2 KO Mice upon Bronchoconstriction

**Figure 6.**
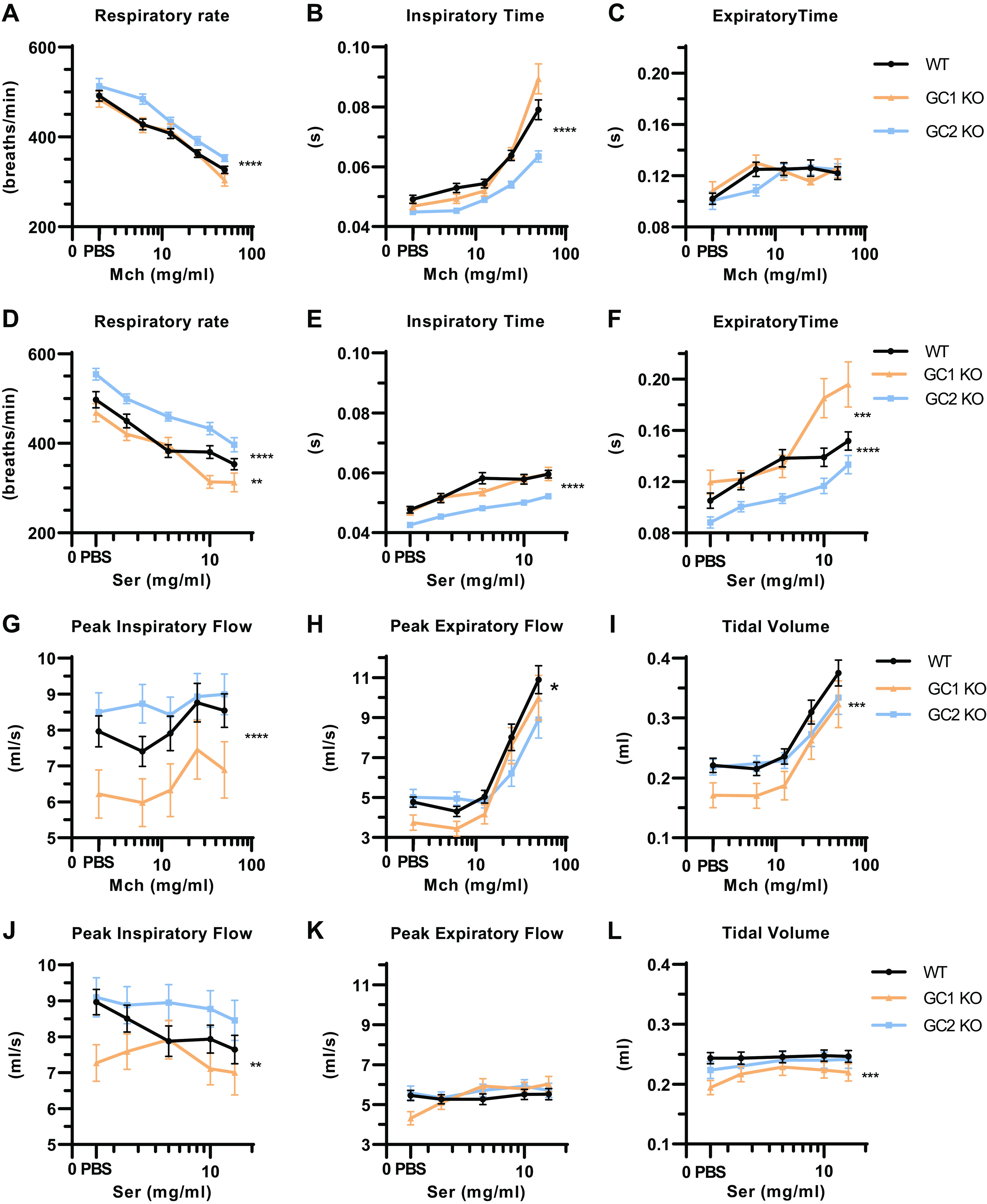
Respiratory parameters of conscious NO-GC1 and NO-GC2 KO mice in response to bronchoconstriction. Respiratory parameters of conscious WT, NO-GC1, and NO-GC2 KO mice challenged with Mch and serotonin on different days and interchangeable order were assessed by unrestrained whole body plethysmography. Respiratory rates (*A* and *D*), times of inspiration (*B* and *E*) and expiration (*C* and *F*), peak inspiratory (*G* and *J*) and expiratory flows (*H* and *K*), and tidal volumes (*I* and *L*) are represented. Averaged value means ± SE of 33 WT, 18 NO-GC1 KO, and 20 NO-GC2 KO mice are shown. The *y*-axis is truncated. Statistical significance was determined by two-way ANOVA. ****P < 0.0001, ***P < 0.001, **P < 0.01, and *P < 0.05. KO, knockout; Mch, methacholine; NO-GC, nitric oxide-sensitive guanylyl cyclase; PBS, phosphate-buffered saline; Ser, serotonin; WT, wild type.

Respiratory rates decreased dose-dependently in response to inhaled Mch or serotonin, but remained higher in NO-GC2 KOs (Fig. 6, *A* and *D*). Consistent with slower breathing, inspiration and expiration times were extended by either bronchoconstrictor with prolongation in NO-GC2 KO mice being less than in WT (Fig. 6, *B, E*, and *F*).

Inhaled Mch did not change the peak inspiratory flow (Fig. 6*G*), whereas peak expiratory flow (Fig. 6*H*) and tidal volume (Fig. 6*I*) were increased by Mch in all genotypes. Minute ventilation—a product of respiratory rate and tidal volume—was maintained unchanged under Mch provocation (Supplemental Fig. S2*A*). Inhaled serotonin decreased peak inspiratory flow only in WT (Fig. 6*J*), while peak expiratory flow (Fig. 6*K*) and tidal volume (Fig. 6*L*) remained unchanged in all genotypes. Minute ventilation decreased with inhaled serotonin (Supplemental Fig. S2*B*). Overall, the curves created from the measurements during Mch or serotonin inhalation were lower in NO-GC1 KO and WT-like in NO-GC2 KO mice.

### Conscious NO-GC1 and NO-GC2 KO Mice Display Opposite Alterations of Airway Responsiveness

**Figure 7.**
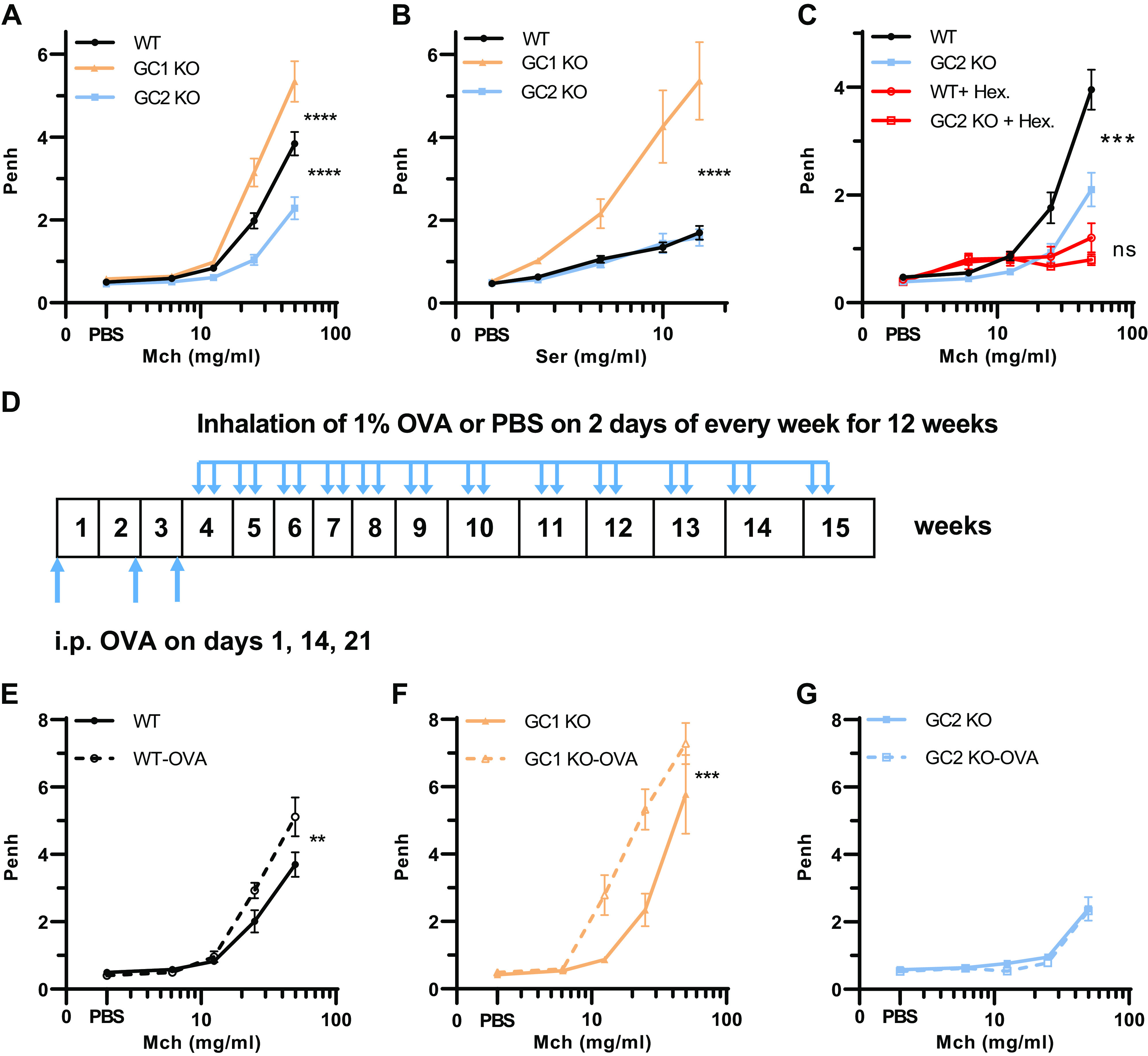
Airways hyperresponsiveness is enhanced in NO-GC1 KO and reduced in NO-GC2 KO mice. The enhanced pause (Penh) of conscious WT (*n* = 33), NO-GC1 KO (*n* = 18), and NO-GC2 KO (*n* = 20) mice challenged with Mch (*A*) and serotonin (*B*) was assessed by unrestrained whole body plethysmography. *C*: the Penh in response to Mch was measured in WT (*n* = 10) and NO-GC2 KO mice (*n* = 11) before and after application of the ganglion blocker hexamethonium (30 mg/kg ip) 2 days apart. *D*: mice were immunized three times with OVA (20 µg ip in Alum) and challenged with nebulized OVA (1%) or PBS on two consecutive days of every week for 12 wk to induce airway hyperresponsiveness. Plethysmography measurements were performed 1 day after the last challenge with OVA/PBS. *E*–*G*: the averaged Penh value means ± SE of OVA-immunized and OVA-challenged mice are shown compared with the corresponding OVA-immunized and PBS-challenged controls [WT (*n* = 13), NO-GC1 KO (*n* = 7), and NO-GC2 KO (*n* = 6)]. Statistical significance was determined by two-way ANOVA. ****P < 0.0001, ***P < 0.001, and **P < 0.01; ns, not significant. KO, knockout; Mch, Methacholine; NO-GC, nitric oxide-sensitive guanylyl cyclase; OVA, ovalbumin; PBS, phosphate-buffered saline; Ser, serotonin; WT, wild type.

A widely reported parameter of the unrestrained whole body plethysmography is Penh, i.e., enhanced pause, a composite, dimensionless value (Penh = PEF/PIF × [(Te/RT)-1]) that was taken as an indicator of airway responsiveness. The Penh of NO-GC1 KOs inhaling Mch or serotonin was higher compared with WT, which is consistent with the increased resistance measured in these mice (Fig. 7, *A* and *B*). The NO-GC2 KOs displayed a lower Penh with Mch and WT-like with serotonin, indicating that the smooth muscle function of their airways is not generally impaired (Fig. 7, *A* and *B*).

Mice were measured 2 days apart before and after the application of the ganglion blocker hexamethonium (30 mg/kg ip) to determine whether an altered vagal input can account for the reduced response to Mch in NO-GC2 KOs. The increase of Penh by Mch was reduced in mice treated with hexamethonium and no longer differed between WT and NO-GC2 KOs (Fig. 7*C*). The greater effect of the ganglion blocker in WT compared with NO-GC2 KOs is compatible with the assumption of a reduced vagal tonus in the latter.

We applied an OVA model of asthma known to induce airway hyperresponsiveness to examine further the diametrically opposed responses to Mch in the lungs of NO-GC1 KO and NO-GC2 KO mice (Fig. 7*D*). The Penh, in response to Mch, increased in OVA-challenged WT (Fig. 7*E*) and NO-GC1 KO mice (Fig. 7*F*) compared with their respective controls, but remained unchanged in OVA-challenged NO-GC2 KO mice (Fig. 7*G*). Moreover, the increase was more pronounced in OVA-challenged NO-GC1 KOs than in WT (increase at 50 mg/mL Mch in %: GC1 KO, 1559 ± 140 vs, WT 1288 ± 135, *P* = 0.011, two-sided *t* test).

We next examined whether the lack of OVA-induced airway reactivity elicited by Mch in NO-GC2 KO mice was caused by a failure to immunologically sensitize the mice. Therefore, we counted and differentiated the leucocytes (i.e., macrophages, lymphocytes, eosinophils, and neutrophils) obtained by bronchoalveolar lavage. The OVA challenge increased the numbers of immune cells in the lungs of NO-GC2 KO and WT mice to a similar degree, suggesting that the loss of airway reactivity observed in the NO-GC2 KO mice was not caused by a decrease in lung inflammation (Supplemental Fig. S3, *A*–*E*).

## DISCUSSION

A knowledge gap exists, so far, regarding the individual roles of the NO-GC1 and NO-GC2 in the lung. The present study examined the pulmonary function of mice lacking either one of the two enzymes signaling by forming cGMP in response to NO. The results acquired from invasive and noninvasive measurements suggest that both NO-GC isoforms participate in the regulation of airway smooth muscle function via distinct pathways.

The NO-GC1 was identified as the major isoform responsible for NO-induced cGMP formation in murine lung, whereas NO-GC2 contributes only 10% to the overall NO-GC activity, a finding confirming previous results from our and other groups (20, 28, 29). The PDE activities responsible for cGMP degradation were dominated by PDE5 and were not reduced in either NO-GC KO strain, suggesting no altered PDE5 expression. We demonstrated the cGMP synthesis by NO-GC2 in lung tissue in a cellular context for the first time by measuring the cGMP increases in response to NO (GSNO 100 µM, 5 min) while PDE5 was inhibited with sildenafil. Concomitantly, we revealed the impact of cGMP degradation over synthesis, because the same NO concentration in the presence of an inhibited PDE5 resulted in comparable cGMP levels in lung slices of WT and KO mice of NO-GC1 or NO-GC2. Additional mechanisms in recent studies have been proposed that can regulate NO/cGMP signaling by triggering processes that promote the inactivation of NO-GCs or assist in their recovery (30).

In our experiments, we used a high concentration of GSNO as NO-supplying agent, because it slowly releases NO, and thereby, an ongoing NO presence can be maintained over the experimental duration (5 min). However, it must be mentioned that GSNO (*S*-nitroso-glutathione) is endogenously present in the lungs at nanomolar levels and exerts smooth muscle effects in a primarily NO-independent manner (6). Regarding human lung physiology, GSNO is referred to as an endogenous bronchodilator, whereas NO itself is not able to induce relaxation in human airways at normal airway concentrations (∼20 ppb) and clinically does not cause bronchodilation (4, 6). Clinical effects of NO/cGMP signaling in the lung are primarily pulmonary vasodilation and anti-inflammatory responses by preventing adherence and activation of immune cells and platelets.

The absence of cGMP formed by NO-GC1 or NO-GC2 resulted in biochemical changes that affected lung mechanical properties differentially.

The abrogation of NO-GC1-derived cGMP resulted in an enhanced resistance of the respiratory system (*R*_rs_) that is most probably caused by enhanced smooth muscle shortening because of the eliminated relaxing pathway. Airway resistance (*R*_n_), which mainly represents the larger proximal airways, was unaltered under nonstimulated conditions. However, tissue resistance (*G*), a parameter indicating parenchymal distortion that can occur by airways being constricted, was enhanced in NO-GC1 KO mice, in support of the notion of airways in the lung periphery of these mice being narrowed. As further consequences, inspiratory capacity in NO-GC1 KO mice was reduced, whereas elastance (*E*) and elastic recoil were confirmed to be higher accordingly. Together, these findings point to resistive lung properties of the NO-GC1 KO mice resembling those displayed by mice with fibrosis (31, 32). Consistent with this, the Mch increased the resistance of the respiratory system (*R*_rs_) and the airways (*R*_n_) of the NO-GC1 KO mice to a higher degree compared with WT, an effect that could be normalized by a concomitant application of a bronchodilator. The higher response to Mch assigns NO-GC1 a role in counteracting the narrowing of the airways. Moreover, it suggests an ongoing participation of NO-GC1 in adjusting the airway smooth muscle tone, similar to that established for vascular tone, and implies enhanced NO generation during airway constriction.

Different from the NO-GC1 deficiency, the absence of the NO-GC2 does not affect parameters related to resistance, but results in a lower tissue elasticity (*H* value) and an upward shift of the pressure-volume loops toward higher lung volumes, both indicating a reduced elastic recoil in NO-GC2 KO lungs. Hence, alveolar spaces of NO-GC2 KO mice tend to become more distended by applying an external mechanical force and, indeed, histological analysis revealed enlarged airspaces in the KO lungs fixed by a constant pressure. The lung tissue parameters of the NO-GC2 KO mice generally show many similarities to mice with elastase-induced emphysema (31, 32). However, NO-GC2 KO mice did not display any decreased airway resistance (*R*_n_), and Mch provocation affected their lung functional parameters to a lower degree than WT mice.

The hyporesponsiveness to Mch may point to a procontractile role for NO-GC2. However, this seems a paradox, because NO-GC2 as NO-GC1 is known to mediate smooth muscle relaxation (20, 33, 34). A general impairment of the airways can be largely excluded because serotonin provocation induced a WT-like response. We assumed that the hyporesponsiveness to Mch is most probably a consequence of a reduced cholinergic input in airways because of altered local reflexes. Compatible with this assumption, the response to Mch was reduced by a ganglion blocker, with the effect being greater in WT compared with NO-GC2 KO mice. Cholinergic input is known to modify the contractile ability of airway smooth muscle cells and has been shown to depend mainly on afferent nerve activity arising from intrapulmonary airways and lungs (35, 36). Since the Mch effect was obvious in both mechanically ventilated and conscious mice, a modified cholinergic input because of altered central mechanisms can be widely excluded.

Afferent nerves innervating airways’ smooth muscles are involved in vagal reflexes influencing breathing. Conversely, many stimuli that have been shown to induce changes in respiration alter the smooth muscle tone coincidently by modifying the activity of the parasympathetic input (37, 38). In support of altered vagal reflexes, conscious NO-GC2 KO mice displayed altered breathing patterns at rest and after challenge of the mice with a bronchoconstrictor.

The opposite responsiveness to Mch observed in mice lacking either NO-GC1 or NO-GC2 also persisted after the OVA challenge of the mice. More precisely, responsiveness to Mch was further enhanced by OVA challenge in NO-GC1 KO mice, whereas no increase was observed in NO-GC2 KO mice. The increased airway reactivity by OVA challenge in WT mice is known to correlate with enhanced lung inflammation. Nevertheless, the processes underlying the hyper- or hyporesponsiveness to Mch shown in the two KO strains cannot be explained by different immune responses. We showed in a previous study that NO-GC1 KO mice developed a rather attenuated type 2 inflammation in the OVA model, which resulted in fewer morphological changes (27). Likewise, we showed here that lung inflammation in NO-GC2 KO mice was increased similarly to that in the OVA-challenged WT controls.

Increased oxidative and nitrosative stress that is present in the inflamed airways in the asthmatic lung has been reported to damage NO-GCs, whereby a portion of the enzymes becomes less sensitive or insensitive to NO stimulation (39). In addition, a dysregulated balance of further *S*-nitrosylated protein targets has also been implicated in the asthma pathophysiology (40). Such alterations have also most probably taken place in our OVA-challenged mice; however, we see no evidence that they could have progressed to different degrees between the WT and KO mice.

Similar to the KO mice of NO-GC1 or NO-GC2, eNOS KO mice have been shown to exhibit increased (41) and nNOS KOs reduced responsiveness to inhaled Mch under normal conditions (42) and even after OVA challenge (43). In addition, nNOS KO mice exhibited augmented respiratory responses to hypoxia (44). Thus, it is tempting to speculate about eNOS and nNOS as sources of NO that regulate NO-GC1 and NO-GC2 activity, respectively. This is a concept that needs to be addressed in forthcoming studies.

Detailed mechanisms underlying the functional roles of NO-GC isoforms in airways cannot be determined from the data acquired in the present study. Similarly, an altered contribution of other physiological pathways to our results cannot be excluded. Nevertheless, a novel concept regarding NO/cGMP signaling in airways can be drawn. Accordingly, two distinct NO/cGMP pathways exist, which are differentially engaged in the regulation of airway smooth muscle function: A relaxing pathway mediated by NO-GC1, which acts to limit the extent of muscle shortening, and a second one mediated by NO-GC2, that adjusts airway responsiveness most probably by influencing parasympathetic input. Differential targeting of these pathways could provide novel therapeutic strategies for treating obstructive lung diseases.

## DATA AVAILABILITY

Data will be made available upon reasonable request.

## SUPPLEMENTAL DATA

10.6084/m9.figshare.19771723 Supplemental Figs. S1–S3: https://doi.org/10.6084/m9.figshare.19771723.

## GRANTS

This work was supported by a grant from the Dr. Georg E. und Marianne Kosing Foundation.

## DISCLOSURES

No conflicts of interest, financial or otherwise, are declared by the author(s).

## AUTHOR CONTRIBUTIONS

M.P. and E.M. conceived and designed research; M.V., M.P., S.G., M.P., and E.M. performed experiments; M.V., M.P., and E.M. analyzed data; M.V. and E.M. interpreted results of experiments; E.M. prepared figures; E.M. drafted manuscript; M.V., M.P., D.K., M.P., and E.M. edited and revised manuscript; M.V., M.P., S.G., D.K., M.P., and E.M. approved final version of manuscript.

## References

[1] Schrammel A, Behrends S, Schmidt K, Koesling D, Mayer B. Characterization of 1H-[1,2,4]oxadiazolo[4,3-a]quinoxalin-1-one as a heme-site inhibitor of nitric oxide-sensitive guanylyl cyclase. Mol Pharmacol 50: 1–5, 1996. 8700100

[2] Wittenborn EC, Marletta MA. Structural perspectives on the mechanism of soluble guanylate cyclase activation. Int J Mol Sci 22: 5439, 2021. doi:10.3390/ijms22115439. 34064029 PMC8196705

[3] Sandner P, Vakalopoulos A, Hahn MG, Stasch JP, Follmann M. Soluble guanylate cyclase stimulators and their potential use: a patent review. Expert Opin Ther Pat 31: 203–222, 2021. doi:10.1080/13543776.2021.1866538. 33395323

[4] Ricciardolo FL, Sterk PJ, Gaston B, Folkerts G. Nitric oxide in health and disease of the respiratory system. Physiol Rev 84: 731–765, 2004. doi:10.1152/physrev.00034.2003. 15269335

[5] Perez-Zoghbi JF, Bai Y, Sanderson MJ. Nitric oxide induces airway smooth muscle cell relaxation by decreasing the frequency of agonist-induced Ca^2+^ oscillations. J Gen Physiol 135: 247–259, 2010. doi:10.1085/jgp.200910365. 20176853 PMC2828908

[6] Marozkina NV, Gaston B. Nitrogen chemistry and lung physiology. Annu Rev Physiol 77: 431–452, 2015. doi:10.1146/annurev-physiol-021113-170352. 25668023

[7] Sanna A, Kurtansky A, Veriter C, Stănescu D. Bronchodilator effect of inhaled nitric oxide in healthy men. Am J Respir Crit Care Med 150: 1702–1704, 1994. doi:10.1164/ajrccm.150.6.7952636. 7952636

[8] Koziol-White CJ, Ghosh A, Sandner P, Erzurum SE, Stuehr DJ, Panettieri RA. Jr. Soluble guanylate cyclase agonists induce bronchodilation in human small airways. Am J Respir Cell Mol Biol 62: 43–48, 2020. doi:10.1165/rcmb.2019-0001OC. 31340135 PMC6938135

[9] Dupont LL, Glynos C, Bracke KR, Brouckaert P, Brusselle GG. Role of the nitric oxide-soluble guanylyl cyclase pathway in obstructive airway diseases. Pulm Pharmacol Ther 29: 1–6, 2014. doi:10.1016/j.pupt.2014.07.004. 25043200

[10] Ghosh A, Koziol-White CJ, Asosingh K, Cheng G, Ruple L, Groneberg D, Friebe A, Comhair SA, Stasch JP, Panettieri RA, Jr, Aronica MA, Erzurum SC, Stuehr DJ. Soluble guanylate cyclase as an alternative target for bronchodilator therapy in asthma. Proc Natl Acad Sci USA 113: E2355–E2362, 2016. doi:10.1073/pnas.1524398113. 27071111 PMC4855555

[11] Lam M, Bourke JE. A new pathway to airway relaxation: targeting the “other” cyclase in asthma. Am J Respir Cell Mol Biol 62: 3–4, 2020. doi:10.1165/rcmb.2019-0274ED. 31414885 PMC6938138

[12] Ricciardolo FL. Multiple roles of nitric oxide in the airways. Thorax 58: 175–182, 2003. doi:10.1136/thorax.58.2.175. 12554905 PMC1746564

[13] Ghofrani HA, Grimminger F. Modulating cGMP to treat lung diseases. Handb Exp Pharmacol 191: 469–483, 2009. doi:10.1007/978-3-540-68964-5_20. PMC7121669 19089341

[14] Ward JK, Belvisi MG, Fox AJ, Miura M, Tadjkarimi S, Yacoub MH, Barnes PJ. Modulation of cholinergic neural bronchoconstriction by endogenous nitric oxide and vasoactive intestinal peptide in human airways in vitro. J Clin Invest 92: 736–742, 1993. doi:10.1172/JCI116644. 8349813 PMC294908

[15] Kesler BS, Mazzone SB, Canning BJ. Nitric oxide-dependent modulation of smooth-muscle tone by airway parasympathetic nerves. Am J Respir Crit Care Med 165: 481–488, 2002. doi:10.1164/ajrccm.165.4.2004005. 11850340

[16] Corbin JD, Beasley A, Blount MA, Francis SH. High lung PDE5: a strong basis for treating pulmonary hypertension with PDE5 inhibitors. Biochem Biophys Res Commun 334: 930–938, 2005. doi:10.1016/j.bbrc.2005.06.183. 16023993

[17] Russwurm M, Behrends S, Harteneck C, Koesling D. Functional properties of a naturally occurring isoform of soluble guanylyl cyclase. Biochem J 335: 125–130, 1998. doi:10.1042/bj3350125. 9742221 PMC1219760

[18] Mergia E, Russwurm M, Zoidl G, Koesling D. Major occurrence of the new alpha2beta1 isoform of NO-sensitive guanylyl cyclase in brain. Cell Signal 15: 189–195, 2003. doi:10.1016/s0898-6568(02)00078-5. 12464390

[19] Friebe A, Mergia E, Dangel O, Lange A, Koesling D. Fatal gastrointestinal obstruction and hypertension in mice lacking nitric oxide-sensitive guanylyl cyclase. Proc Natl Acad Sci USA 104: 7699–7704, 2007. doi:10.1073/pnas.0609778104. 17452643 PMC1863512

[20] Mergia E, Friebe A, Dangel O, Russwurm M, Koesling D. Spare guanylyl cyclase NO receptors ensure high NO sensitivity in the vascular system. J Clin Invest 116: 1731–1737, 2006. doi:10.1172/JCI27657. 16614755 PMC1435723

[21] Stegbauer J, Friedrich S, Potthoff SA, Broekmans K, Cortese-Krott MM, Quack I, Rump LC, Koesling D, Mergia E. Phosphodiesterase 5 attenuates the vasodilatory response in renovascular hypertension. PLoS One 8: e80674, 2013. doi:10.1371/journal.pone.0080674. 24260450 PMC3829872

[22] Jäger R, Groneberg D, Lies B, Bettaga N, Kümmel M, Friebe A. Radioimmunoassay for the quantification of cGMP levels in cells and tissues. Methods Mol Biol 1020: 63–72, 2013. doi:10.1007/978-1-62703-459-3_4. 23709026

[23] Hartney JM, Robichaud A. Assessment of airway hyperresponsiveness in mouse models of allergic lung disease using detailed measurements of respiratory mechanics. Methods Mol Biol 1032: 205–217, 2013. doi:10.1007/978-1-62703-496-8_16. 23943455

[24] Robichaud A, Fereydoonzad L, Limjunyawong N, Rabold R, Allard B, Benedetti A, Martin JG, Mitzner W. Automated full-range pressure-volume curves in mice and rats. J Appl Physiol (1985) 123: 746–756, 2017. doi:10.1152/japplphysiol.00856.2016. 28751375 PMC5668446

[25] Rasband WS. ImageJ; National Institutes of Health: Bethesda, MD, USA. http://imagej.nih.gov/ij/ (accessed on 29 November 2017).

[26] Crowley G, Kwon S, Caraher EJ, Haider SH, Lam R, Batra P, Melles D, Liu M, Nolan A. Quantitative lung morphology: semi-automated measurement of mean linear intercept. BMC Pulm Med 19: 206, 2019. doi:10.1186/s12890-019-0915-6. 31706309 PMC6842138

[27] Gnipp S, Mergia E, Puschkarow M, Bufe A, Koesling D, Peters M. Nitric oxide dependent signaling via cyclic GMP in dendritic cells regulates migration and T-cell polarization. Sci Rep 8: 10969, 2018. doi:10.1038/s41598-018-29287-9. 30030528 PMC6054623

[28] Buys ES, Sips P, Vermeersch P, Raher MJ, Rogge E, Ichinose F, Dewerchin M, Bloch KD, Janssens S, Brouckaert P. Gender-specific hypertension and responsiveness to nitric oxide in sGCalpha1 knockout mice. Cardiovasc Res 79: 179–186, 2008. doi:10.1093/cvr/cvn068. 18339647

[29] Bachiller PR, Cornog KH, Kato R, Buys ES, Roberts JD. Jr.,Soluble guanylate cyclase modulates alveolarization in the newborn lung. Am J Physiol Lung Cell Mol Physiol 305: L569–L581, 2013. doi:10.1152/ajplung.00401.2012. 23934926 PMC3798773

[30] Stuehr DJ, Misra S, Dai Y, Ghosh A. Maturation, inactivation, and recovery mechanisms of soluble guanylyl cyclase. J Biol Chem 296: 100336, 2021. doi:10.1016/j.jbc.2021.100336. 33508317 PMC7949132

[31] Vanoirbeek JA, Rinaldi M, De Vooght V, Haenen S, Bobic S, Gayan-Ramirez G, Hoet PH, Verbeken E, Decramer M, Nemery B, Janssens W. Noninvasive and invasive pulmonary function in mouse models of obstructive and restrictive respiratory diseases. Am J Respir Cell Mol Biol 42: 96–104, 2010. doi:10.1165/rcmb.2008-0487OC. 19346316

[32] Devos FC, Maaske A, Robichaud A, Pollaris L, Seys S, Lopez CA, Verbeken E, Tenbusch M, Lories R, Nemery B, Hoet PH, Vanoirbeek JA. Forced expiration measurements in mouse models of obstructive and restrictive lung diseases. Respir Res 18: 123, 2017. doi:10.1186/s12931-017-0610-1. 28629359 PMC5477381

[33] Nimmegeers S, Sips P, Buys E, Brouckaert P, Van de Voorde J. Functional role of the soluble guanylyl cyclase alpha(1) subunit in vascular smooth muscle relaxation. Cardiovasc Res 76: 149–159, 2007. doi:10.1016/j.cardiores.2007.06.002. 17610859

[34] Mergia E, Thieme M, Hoch H, Daniil G, Hering L, Yakoub M, Scherbaum CR, Rump LC, Koesling D, Stegbauer J. Impact of the NO-sensitive guanylyl cyclase 1 and 2 on renal blood flow and systemic blood pressure in mice. Int J Mol Sci 19: 967, 2018. doi:10.3390/ijms19040967. PMC5979494 29570672

[35] Jammes Y, Mei N. Assessment of the pulmonary origin of bronchoconstrictor vagal tone. J Physiol. 291: 305–316, 1979. doi:10.1113/jphysiol.1979.sp012814. 480218 PMC1280902

[36] Kesler BS, Canning BJ. Regulation of baseline cholinergic tone in guinea-pig airway smooth muscle. J Physiol 518: 843–855, 1999. doi:10.1111/j.1469-7793.1999.0843p.x. 10420019 PMC2269456

[37] Canning BJ. Reflex regulation of airway smooth muscle tone. J Appl Physiol (1985) 101: 971–985, 2006. doi:10.1152/japplphysiol.00313.2006. 16728519

[38] Mazzone SB, Undem BJ. Vagal afferent innervation of the airways in health and disease. Physiol Rev 96: 975–1024, 2016. doi:10.1152/physrev.00039.2015. 27279650 PMC4982036

[39] Ghosh A, Koziol-White CJ, Jester WF, Jr, Erzurum SC, Asosingh K, Panettieri RA, Jr, Stuehr DJ. An inherent dysfunction in soluble guanylyl cyclase is present in the airway of severe asthmatics and is associated with aberrant redox enzyme expression and compromised NO-cGMP signaling. Redox Biol 39: 101832, 2021. doi:10.1016/j.redox.2020.101832. 33360351 PMC7772568

[40] Foster MW, Hess DT, Stamler JS. Protein S-nitrosylation in health and disease: a current perspective. Trends Mol Med 15: 391–404, 2009. doi:10.1016/j.molmed.2009.06.007. 19726230 PMC3106339

[41] Feletou M, Lonchampt M, Coge F, Galizzi JP, Bassoullet C, Merial C, Robineau P, Boutin JA, Huang PL, Vanhoutte PM, Canet E. Regulation of murine airway responsiveness by endothelial nitric oxide synthase. Am J Physiol Lung Cell Mol Physiol 281: L258–L267, 2001. doi:10.1152/ajplung.2001.281.1.L258. 11404269

[42] De Sanctis GT, Mehta S, Kobzik L, Yandava C, Jiao A, Huang PL, Drazen JM. Contribution of type I NOS to expired gas NO and bronchial responsiveness in mice. Am J Physiol 273: L883–L888, 1997. doi:10.1152/ajplung.1997.273.4.L883. 9357865

[43] De Sanctis GT, MacLean JA, Hamada K, Mehta S, Scott JA, Jiao A, Yandava CN, Kobzik L, Wolyniec WW, Fabian AJ, Venugopal CS, Grasemann H, Huang PL, Drazen JM. Contribution of nitric oxide synthases 1, 2, and 3 to airway hyperresponsiveness and inflammation in a murine model of asthma. J Exp Med 189: 1621–1630, 1999. doi:10.1084/jem.189.10.1621. 10330441 PMC2193630

[44] Kline DD, Yang T, Huang PL, Prabhakar NR. Altered respiratory responses to hypoxia in mutant mice deficient in neuronal nitric oxide synthase. J Physiol 511: 273–287, 1998. doi:10.1111/j.1469-7793.1998.273bi.x. 9679181 PMC2231102

